# Genome Anchored QTLs for Biomass Productivity in Hybrid *Populus* Grown under Contrasting Environments

**DOI:** 10.1371/journal.pone.0054468

**Published:** 2013-01-29

**Authors:** Wellington Muchero, Mitchell M. Sewell, Priya Ranjan, Lee E. Gunter, Timothy J. Tschaplinski, Tongming Yin, Gerald A. Tuskan

**Affiliations:** 1 Bioscience Division, Oak Ridge National Laboratory, Oak Ridge, Tennessee, United States of America; 2 BioEnergy Science Center, Oak Ridge National Laboratory, Oak Ridge, Tennessee, United States of America; University of Guelph, Canada

## Abstract

Traits related to biomass production were analyzed for the presence of quantitative trait loci (QTLs) in a *Populus trichocarpa* × *P. deltoides* F_2_ population. A genetic linkage map composed of 841 SSR, AFLP, and RAPD markers and phenotypic data from 310 progeny were used to identify genomic regions harboring biomass QTLs. Twelve intervals were identified, of which *BM-1*, *BM-2*, and *BM-7* were identified in all three years for both height and diameter. One putative QTL, *BM-7,* and one suggestive QTL exhibited significant evidence of over-dominance in all three years for both traits. Conversely, QTLs *BM-4* and *BM-6* exhibited evidence of under-dominance in both environments for height and diameter. Seven of the nine QTLs were successfully anchored, and QTL peak positions were estimated for each one on the *P. trichocarpa* genome assembly using flanking SSR markers with known physical positions. Of the 3,031 genes located in genome-anchored QTL intervals, 1,892 had PFAM annotations. Of these, 1,313, representing 255 unique annotations, had at least one duplicate copy in a QTL interval identified on a separate scaffold. This observation suggests that some QTLs identified in this study may have shared the same ancestral sequence prior to the salicoid genome duplication in *Populus*.

## Introduction

Hybrid poplars have been intensively cultivated in North America as a short-rotation woody crop species for bioenergy and pulp and paper industries [1], [2], [3], [4]. The recent focus on lignocellulosic biofuels from plant biomass as a complement to fossil fuels has led to increased interest in the genetic characteristics of *Populus* as a rapidly growing biomass feedstock [5]. Several factors account for *Populus* success as a feedstock for biofuels and pulp and paper industries, foremost of which is the interspecific hybridization resulting in hybrids with marked improvement in performance, hybrid vigor (i.e heterosis or over-dominance) compared to parental genotypes [6]. Being a genetically diverse genus which displays considerable variation among its species in such adaptive traits as stem growth, crown architecture, and disease resistance, the genetic diversity can be captured through the ease of hybridization among the approximately 30 species of *Populus*. The most desirable clones from these hybrid combinations can then be easily propagated by the well-developed vegetative systems inherent to *Populus*. Inter-american hybrids [7] generated from crosses between *Populus trichocarpa* Torr. & Gray and *Populus deltoides* Bartr. ex Marsh. (*i.e.*, T×D hybrids) are estimated to produce as much as 35 Mg•ha^−1^•yr^−1^ of aboveground biomass at age four [8]. Much of the success of the T×D hybrids is thought to result from the complementary combination of desirable traits inherited from each of the parental species in conjunction with the associated hybrid vigor [9]. Although hybrids exhibit superior performance at the overall phenotypic level, out-breeding depression or under-dominance, at the individual locus level resulting from combining alleles that result in poorer performance of the hybrid relative to parental genotypes is also a known genetic phenomenon [10], and may prevent hybrids from achieving maximum possible performance. Therefore, it is of primary interest to identify specific genomic loci contributing toward such hybrid vigor as well as those that contribute toward out-breeding depression for marker-assisted pyramiding of beneficial loci.

Biomass productivity traits are generally quantitative in nature involving numerous genes and genetic pathways whose activity may be modified by the environment leading, sometimes, to environment specific trait expression [11]. Understanding the genotype x environmental interactions of these genetic elements may enable targeted introgression of beneficial loci through ideotype breeding strategies [12]. Numerous studies have identified quantitative trait loci (QTLs) that are involved in biomass accumulation using hybrid *Populus* pedigrees. Results of these studies were generally reproducible among different studies and environments [13]–[17] suggesting that economically beneficial genes can be isolated for *Populus* improvement.

With the first genome sequence of any tree species [18] in addition to a well developed DNA molecular marker resource, *Populus* has mature genomic resources that should allow for in-depth characterization of loci of interest. Although QTL intervals typically include tens or hundreds of genes, candidate gene mapping has provided some insight into potentially valuable gene targets for improving hybrid *Populus* production. In addition, prioritization of marker saturation can be accurately guided by knowing the physical interval compared to using cM distances whose relationship with physical distance is not always linear due to the heterogeneity of recombination rates across the *Populus* genome [19]. To date, numerous strategies have been applied in mapping efforts to identify potentially viable loci down to the gene level. These strategies include differential gene content in syntenic genomic regions where QTL presence or absence may suggest genetic determinants of economically important traits [20].

The overarching objective of this study was to build on previous work that identified and characterized genetically-driven variation in growth phenotypes within an F_2_ *P. trichocarpa* × *P. deltoides* pedigree. In this work, estimates of QTL numbers [12], QTL positions on a genetic linkage map [14], and aspects related to hybrid vigor, genotype-by-environment interaction (G×E), genetic correlations, and broad-sense heritability at the phenotypic level [15] were characterized. However, due to the molecular anonymity of RFLP, STS, and RAPD markers used in these studies and the unavailability of a reference genome at that time, the genomic location and genic features associated with these loci remained anonymous. The completion of the *Populus* genome assembly [18] and the incorporation of SSR markers with known physical positions in the genetic map [21] now provide an opportunity for further characterization of loci described in the precedent work.

Therefore, the goals of this study were to reanalyze phenotypic data for the F_2_ pedigree to map QTLs segregating for stem height and diameter using an updated genetic map that incorporated SSR markers with known physical positions. In addition, we sought to provide estimates of the role of G×E interactions and hybrid vigor at the individual QTL level. Finally, we sought to utilize flanking SSR markers to delimit genomic intervals that encompass these QTLs thereby enabling the characterization of genic features associated with these loci.

## Results

### QTL Analysis and Detection Across Contrasting Environments

**Figure 1:**
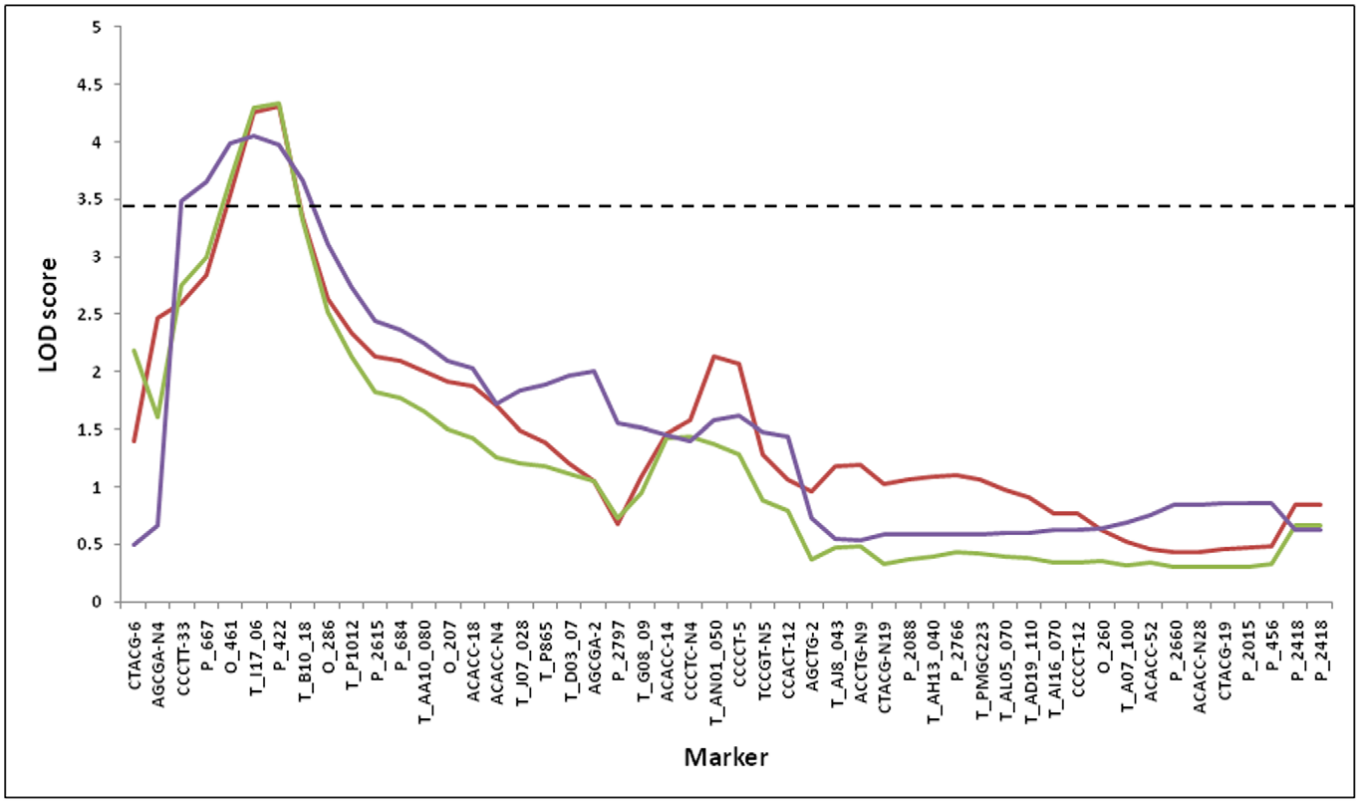
LOD traces for QTL B*M-2* on LG II based on 4-year height (red) and 4-year diameter (green) measured in Clatskanie and 4-year height measured in Boardman (purple). Broken horizontal line represents linkage groupwise LOD significance threshold calculated based on 1,000 permutations at the 0.05 significance level.

Nine putative and three suggestive QTLs were detected for stem height and diameter on eleven linkage groups (LGs) of the family 331 genetic map (Tables 1 and 2). Among the 9 QTLs, 5 were significant in at least one experiment when the more stringent genomewise LOD threshold was used as the cut-off point (Table 1). All QTLs identified in this study were associated with both height and diameter in the same experiment or across different experiments. Of these, six QTLs on LGs I, II, VII, VIII, XIII, and XIV were detected in both Boardman and Clatskanie experimental sites. Three of these on LGs I, II, and XIV were detected in all five datasets analyzed. QTLs *BM-3* and *BM-8* exhibited the highest level of location specificity with QTL x environment interactions showing significance at Prob (>F) <0.1 (Table 3). All QTLs mapped reproducibly in the same map interval and peak positions were typically associated with no more than three markers in close proximity (Table 1). Figure 1 shows a graphical example of QTL *BM-2* and associated peak on LG II highlighting the close agreements between three different phenotypic datasets used to identify the QTL. Averaged across experiments, the percent phenotypic variance explained ranged from 5.2 to 8.5% for each QTL.

**Table 1:** QTLs associated with height and diameter identified in *Populus* family 331 F_2_ pedigree based on linkage-group- (LG) and genome-wise LOD significance thresholds (GW).

QTL (LG)	Marker at QTL peak	LOD score	LOD threshold (LG)	LOD Threshold (GW)	Trait	% PVE	mu(ac)†	mu(ad)‡	mu(bc)‡	mu(bd)†	a	d	d/|a|
*BM-1* (I)	AGCTG-1	3.26	2.9	4.3	Clatskanie 8 yr height	8.1	122.8	117.9	137.4	123.3	−0.25	4.60	18.4
*BM-1* (I)	AGCTG-1	2.52	3.2	4.3	Clatskanie 4 yr height	6.6	8.8	9.8	10.7	8.7	0.05	1.50	30.0
*BM-1* (I)	AGCTG-1	2.33	3.0	4.4	Clatskanie 4 yr diameter	6.6	8.4	10.0	11.2	8.6	−0.10	2.10	21.0
*BM-1* (I)	AGCTG-1	2.51	3.0	4.4	Boardman 4 yr height	5.4	343.7	338.4	381.3	368.2	−12.25	3.90	0.30
*BM-1* (I)	TCCGT-4	2.55	2.9	4.3	Boardman 4 yr diameter	6.8	3.6	3.7	4.3	3.8	−0.10	0.30	3.00
*BM-2* (II)	T_D03_07	3.36	2.8	4.3	Boardman 4 yr diameter	6.0	4.1	3.8	4.1	3.6	0.25	0.10	0.40
*BM-2* (II)	T_I17_06	4.05	3.1	4.4	Boardman 4 yr height	7.2	331.2	383.2	369.9	341.8	−5.30	40.10	7.56
*BM-2* (II)	P_422	4.33	3.0	4.4	Clatskanie 4 yr diameter	7.9	8.4	10.1	11.2	8.2	0.10	2.35	23.50
*BM-2* (II)	P_422	4.31	3.4	4.3	Clatskanie 4 yr height	7.8	8.6	9.9	10.6	8.7	−0.05	1.60	32.00
*BM-2* (II)	CCCTT-33	2.19	2.8	4.3	Clatskanie 8 yr height	5.9	116.5	133.3	127.5	121.0	−2.25	11.65	5.18
*BM-3* (V)	AGCTG-7	7.02	2.7	4.3	Boardman 4 yr diameter	10.5	4.3	3.8	4.0	3.4	0.45	0.05	0.11
*BM-3* (V)	AGCTG-7	3.70	2.9	4.3	Boardman 4 yr height	6.4	374.5	345.8	376.4	329.0	22.75	9.35	0.41
*BM-4* (VII)	T_AE20_120	3.37	2.6	4.3	Boardman 4 yr height	11.6	389.2	355.0	309.2	365	12.10	−45.00	−3.72
*BM-4* (VII)	T_AB14_110	2.76	2.6	4.3	Boardman 4 yr diameter	5.5	4.15	3.8	3.5	3.9	0.12	−0.38	−3.00
*BM-4* (VII)	T_AF04_090	2.62	2.6	4.4	Clatskanie 4 yr diameter	5.1	10.9	9.9	7.8	9.4	0.75	−1.30	−1.73
*BM-4* (VII)	P_2140	2.52	2.6	4.3	Clatskanie 4 yr height	4.2	10.2	10.0	8.3	9.3	0.45	−0.60	−1.33
*BM-5* (VIII)	TCCGT-16	4.94	2.3	4.3	Boardman 4 yr diameter	8.2	4.1	4.1	3.7	3.5	0.30	0.10	0.33
*BM-5* (VIII)	TCCGT-16	2.70	2.5	4.4	Boardman 4 yr height	4.5	370.7	373.2	335.4	346.4	12.15	−4.25	−0.35
*BM-5* (VIII)	TCCGT-16	2.83	2.4	4.3	Clatskanie 8 yr height	5.2	128.7	130.7	116.2	122.0	3.35	−1.90	−0.57
*BM-6* (XIII)	CCCCT-N3	2.83	2.9	4.4	Boardman 4 yr height	6.7	364.6	335.0	347.8	385.8	−10.60	−33.80	−3.19
*BM-6* (XIII)	CCCCT-N3	2.45	2.8	4.3	Boardman 4 yr diameter	5.7	4.0	3.6	3.7	4.2	−0.10	−0.45	−4.50
*BM-6* (XIII)	AGCTG-27	4.26	2.9	4.3	Clatskanie 8 yr height	10.9	121.5	113.6	127.8	136.3	−7.40	−8.20	−1.11
*BM-7* (XIV)	CTACG-N1	4.34	2.7	4.3	Boardman 4 yr diameter	9.0	3.9	4.3	3.9	3.5	0.20	0.40	2.00
*BM-7* (XIV)	AGCGA-14	3.94	2.8	4.4	Boardman 4 yr height	6.7	364.8	370.7	365.2	318.5	23.15	26.30	1.14
*BM-7* (XIV)	AGCGA-14	4.88	2.8	4.4	Clatskanie 4 yr diameter	8.7	9.4	11.3	9.6	7.4	1.00	2.05	2.05
*BM-7* (XIV)	CTACG-N1	3.95	2.8	4.3	Clatskanie 8 yr height	10.5	120.2	132.8	138.8	116.6	1.80	17.4	9.67
*BM-7* (XIV)	CTACG-N1	2.26	2.9	4.3	Clatskanie 4 yr height	5.9	9.4	10.3	10.4	8.5	0.45	1.40	3.11
*BM-8* (XVII)	T_L09_105	2.50	2.4	4.3	Boardman 4 yr diameter	6.2	4.0	4.0	3.5	3.8	0.10	−0.15	−1.50
*BM-8* (XVII)	T_AI06_055	1.94	2.5	4.4	Boardman 4 yr height	4.1	377.3	368.7	342.0	341.8	17.75	−4.2	−0.24
*BM-9* (XIX)	ACCTG-24	3.72	2.7	4.4	Clatskanie 4 yr diameter	10.2	7.9	10.7	9.1	11.5	−1.80	0.20	0.11
*BM-9* (XIX)	ACCTG-24	3.53	2.5	4.3	Clatskanie 4 yr height	11.2	8.3	11.0	9.0	10.5	−1.10	0.60	0.55
*BM-9*(XIX)	AGCTG-8	2.51	2.6	4.3	Clatskanie 8 yr height	3.8	120.5	122.1	124.4	133.5	−6.50	−3.75	−0.58

%PVE = percent phenotypic variance explained;

† Mean associated with heterozygous genotypes ‘ac’ and ‘bd’ where alleles are derived from the same species;

‡ Mean associated with heterozygous genotypes ‘ad’ and ‘bc’ where alleles are derived from different species, a = additive; d = dominance; d/|a| = QTL mode of action.

**Table 2:** Suggestive QTLs associated with height and diameter identified in *Populus* family 331 F_2_ pedigree.

Linkage group	Marker at QTL peak	LOD score	LOD threshold (LG)	Level of significance LG (GW)†	Trait	% PVE	Mu(ac)	Mu(ad)	Mu(bc)	Mu(bd)	a	d	d/|a|
IA	TCCGT-12	2.14	2.9	0.199 (0.777)	Boardman 4 yr diameter	5.8	3.5	3.9	4.2	3.8	−0.15	0.40	2.67
IA	CCCTT-N6	2.49	3.0	0.103 (0.749)	Boardman 4 yr height	5.1	329.2	360.2	381.3	355.7	−13.25	28.30	2.14
IA	CCCTT-N6	2.15	3.0	0.172 (0.940)	Clatskanie 4 yr diameter	5.6	8.2	10.1	11.1	9.0	−0.40	2.00	5.00
IA	CCCTT-N6	2.18	3.2	0.221 (0.955)	Clatskanie 4 yr height	5.7	8.6	10.1	10.5	9.0	−0.20	1.50	7.50
IA	TCCGT-N11	2.55	2.9	0.078 (0.688)	Clatskanie 8 yr height	3.7	120.3	123.4	134.5	122.1	−0.90	7.75	8.60
VI	O_050	2.95	3.0	0.054 (0.472)	Boardman 4 yr diameter	4.4	4.1	3.7	4.1	3.7	0.20	0.00	0.00
VI	O_050	2.61	3.1	0.098 (0.724)	Boardman 4 yr height	4.0	367.6	340.2	376.1	344.4	11.60	2.15	0.19
VI	O_050	2.49	2.9	0.101 (0.755)	Clatskanie 8 yr height	3.8	123.6	119.4	132.7	122.6	0.50	2.95	5.90
XVIII	ACACC-N21	2.66	2.8	0.061 (0.690)	Clatskanie 4 yr diameter	5.3	8.3	11.0	10.0	9.1	−0.40	1.80	4.50
XVIII	O_480	2.47	2.7	0.080 (0.828)	Clatskanie 4 yr height	4.5	8.5	10.3	9.5	9.7	−0.60	0.80	1.33

† LG = Linkage groupwise significance; GW = Genomewise significance.

**Table 3:** AMMI analysis results for location specificity in QTL detection between the Clatskanie and Boardman sites.

QTL	Sum of Squares	F	Prob (>F)
*BM-1*	12.815	4.4956	0.1241
*BM-2*	13.626	3.0333	0.1799
*BM-3*	83.833	7.8745	0.0675
*BM-4*	0.602	0.3765	0.5829
*BM-5*	62.910	3.5125	0.1576
*BM-6*	5.471	0.2690	0.6398
*BM-7*	0.551	0.1091	0.7629
*BM-8*	16.484	8.7336	0.05977
*BM-9*	40.208	2.4787	0.2135

### Additive and Dominant Effects

One putative and one suggestive QTL exhibited consistent evidence of over-dominance across experiments and traits, whereas two putative QTLs exhibited consistent under-dominance across traits and environments (Tables 1 and 2). QTL *BM-7* on LG XIV and a suggestive QTL on LG I were detected in all five datasets and exhibited over-dominance in each case (Tables 1 and 2). Putative QTLs *BM-1* and *BM-2* on LGs I and II, respectively, were also detected in all five datasets but each exhibited over-dominance in four of the five instances (Table1). On the other hand, QTLs *BM-4* and *BM-6* on LGs VII and XIII, respectively, exhibited under-dominance across different environments for both height and diameter (Table 1).

### Genome Anchoring of QTL Intervals

**Figure 2:**
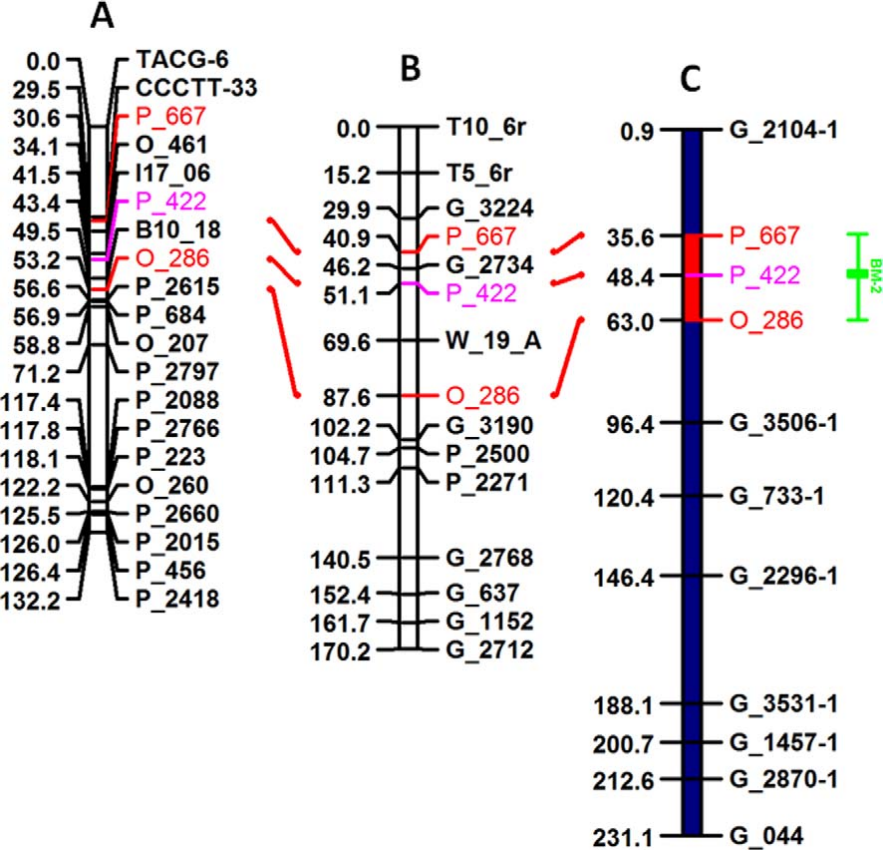
Synteny between (A) Family 331 genetic map LG II, (B) *Populus* consensus genetic map LG II, and (C) Scaffold 2 of the *Populus* genome assembly illustrating the genome anchoring of QTL *BM-2* using flanking markers. Map distance units in A and B represent cM distances and distance units in C represent genomic sequence length (x1OKb).

**Figure 3:**
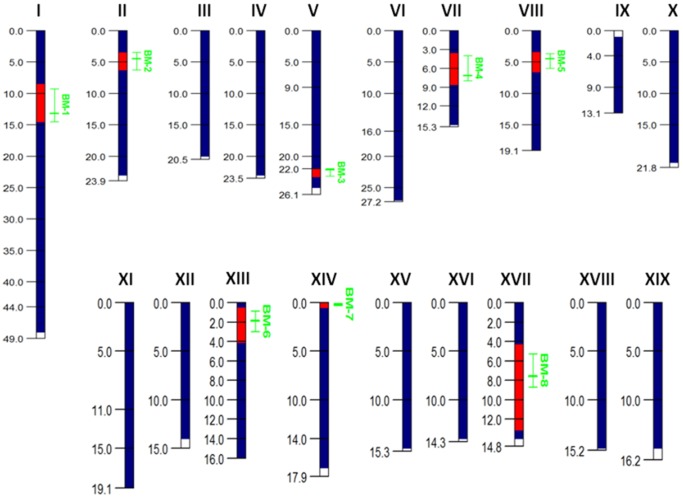
Genome anchored QTL positions on the *Populus* V2.2 assembly. Blue bars represent SSR marker coverage for each scaffold, red bars indicate scaffold intervals between flanking SSR markers used for genome anchoring, vertical green lines represent QTL intervals and estimated peak position. Scaffold intervals are represented in Mb.

In this study, seven of the nine putative QTLs *BM-1*, *BM-2*, *BM-3*, *BM-4*, *BM-5*, *BM-6*, and *BM-8* were successfully anchored on the *Populus* genome assembly (Figures 2 and 3 and Table 4). QTL *BM-9* located on LG XIX could not be anchored due to lack of flanking SSR markers with known physical positions. Figure 2 illustrates the use of two SSR markers flanking a QTL interval peaking at marker P_422 on LG II to anchor and estimate the QTL peak position on the genome assembly. The genome assembly position of QTL *BM-7* on LG XIV was reported previously by Ranjan et al. [20]. Interestingly, both markers associated with *BM-7* on LG XIV, CTACG-N1 and AGCGA-14 (Table 1), were associated with a QTL previously identified for root lignin percentage on the same map position [21]. The lignin QTL, *RL-5*, was subsequently anchored on the genome assembly and analyzed for candidate genes by Ranjan et al. [20]. Further, the QTL exhibited the same pattern of over-dominance for the lignin phenotype as it did for height and diameter in the current study. Genomic intervals covered by individual QTLs ranged from 1.3 to 8.8 Mb (Table 4).

**Table 4:** Genome assembly position for QTLs associated with height and diameter in *Populus* Family F_2_ pedigree.

QTL	Scaffold	Marker at peak	Genome assemblyinterval (Mb)	QTL peak Positionon assembly (Mb)	Interval size (Mb)	Additional SSRs†
*BM-1*	1	AGCTG-1	9.30–14.50	13.10	5.20	35
*BM-2*	2	P_422	3.50–6.30	4.80	2.80	12
*BM-3*	5	AGCTG-7	22.00–23.30	22.20	1.30	12
*BM-4*	7	T_AE20_120	3.60–8.70	7.20	5.10	32
*BM-5*	8	TCCGT-16	3.40–6.60	4.60	3.20	35
*BM-6*	13	CCCCT-N3	0.60–4.10	1.90	3.50	36
*BM-7* ‡	14	AGCGA-14/CTACG-N1	0.01–0.50	0.24	0.49	9
*BM-8*	17	T_L09_105	4.30–13.1	7.50	8.80	32

† See Table S1.

‡ Physical position of the QTL was reported previously by Ranjan et al. (2010).

Based on the QTL intervals defined here and the results of the SSR marker placement on the genome assembly, we identified 197 previously unmapped SSR markers that occurred within QTL intervals identified in this study (Table S1). The number of additional SSR markers ranged from 9 to 36 within individual QTLs (Tables 4 and S1).

### Candidate Gene Identification and Characterization

Intervals spanning the genomic regions summarized in Table 4 were used to identify all genes occurring within the 8 genome-anchored QTLs (Table S2). The number of genes in each interval ranged from 37 for QTL *BM-7* to 721 for QTL *BM-*8. All together, there were 3,031 genes within the 8 genome-anchored QTL intervals. Out of these, 1,892 (62%) had annotations based on PFAM domains and these fell into 290 unique annotations (Table S2). Of the 1,892 annotated gene models, 1,313 (72%) had at least one duplicate in a QTL interval mapping on a different scaffold (Table S3). These represented 255 (88%) of the 290 unique annotations (Table S3).

## Discussion

The genus *Populus* is an economically important tree crop widely grown as feedstock for lignocellulosic biofuels and pulp and paper products, in part, for its rapid growth and ability to thrive in marginal lands that are not suitable for food crop production. Aside from its key importance as an industrial feedstock, *Populus* is also a biological model system for perennial tree crops because of its relatively compact genome, high level of interspecies diversity, and ease of experimental manipulation compared to other genera [4], [18]. Increasing biomass productivity using genetic manipulation is of major interest in contemporary efforts to make biofuels production from *Populus* economically viable. Results presented here provide a basis for the isolation of specific genetic determinants that mediate expression of key productivity traits such as height and diameter. Additionally, this work represents a valuable resource in identification and prioritization of genomic intervals that may be targeted for marker-assisted breeding programs in *Populus*. Below, we discuss specific attributes of this work that should facilitate the application of results presented here in genetic improvement of *Populus* feedstocks.

Given the environmental contrast between the Boardman and Clatskanie experimental sites, QTL detection and expression for the stem-growth traits was remarkably consistent across sites. Only three of the twelve QTLs identified in this study exhibited evidence of location specificity. Therefore, these results indicate that the pattern of phenotypic response from each genotypic class was relatively consistent across the two environments. The generally robust detection of QTL regardless of environment is consistent with findings by Rae et al. [11] who evaluated progeny from the same pedigree in France, United Kingdom, and Italy and identified virtually the same QTL intervals reported in this current study. Given their detection across different geographical environments, QTLs segregating in this pedigree offer valuable targets for further characterization and utilization in improving *Populus* productivity.

Despite the overall hybrid vigor observed for both height and diameter, varying levels of locus-specific QTL mode of action were observed, ranging from under-dominance to over-dominance. The presence of loci exhibiting apparent out-breeding depression (under-dominance) on linkage groups VII and XIII highlight the potential for further improvement of hybrid performance using targeted exclusion of such loci in ideotype breeding approaches. Interestingly, Tschaplinski et al. [10] identified a QTL for osmotic potential exhibiting under-dominance on LG XIII that mapped in the same interval as QTL *BM-6*. Conversely, the QTL peak for *BM-7* on LG XIV, with consistent evidence of over-dominance for both height and diameter, co-located with the same markers which were associated with a root lignin QTL, *RL-5,* which, in a previous study, also exhibited the same over-dominance mode of action [20], [21]. Identification of the same QTL interval and the consistency of QTL mode of action between traits suggest that both height and diameter are largely influenced by the same genes. Such pleiotropic effects have been widely described in *Populus* for both related and diverse traits [12], [20], [21]. Additionally, the association of particular genomic intervals previous associated with phenotypes other than height and diameter suggest that pleiotropy may also exist between apparently non-related traits in *Populus,* although genetic linkage cannot be ruled out based on available data.

Although informative loci have been identified in other studies based on the candidate gene approach in diverse systems such as *Populus* [20], Loblolly pine [22], and cowpeas [23], [24], the large genomic intervals encompassed in some of the QTL intervals can make the narrowing down of candidate genes difficult. This limitation was evident in our analyses where only 1 QTL interval (*BM-7*) encompassed less than 100 genes. Despite this limitation, the unique genome duplication event which resulted in synteny between different *Populus* chromosomal segments [18] provides a reasonable starting point at narrowing down the list of candidate genes. The extensive duplication and paralogous relationship between genes found in QTL intervals located on different chromosomes suggests that these QTLs may have been derived from the same ancestral sequences and is consistent with the high levels of inter-chromosomal synteny described previously by Tuskan et al. [18]. This assumption would imply that functional activity for these genes was conserved post-duplication leading to existence of paralogous QTLs on different *Populus* chromosomes. The opposite scenario, where functional divergence or gene loss occurred after the duplication event resulting in deferential QTL presence on otherwise syntenic chromosome intervals was harnessed to identify unique candidate genes for cell-wall chemistry in a previous study [20]. QTL relationships based on ancestral sequences and candidate gene analyses will benefit from further improvements in the genome assembly and annotation of all gene models. At present, 38% of genes present within the 8 QTL intervals did not have adequate annotation information.

The approach described above, however, does not negate the need for additional marker saturation and fine-mapping of these intervals before candidate genes are selected for molecular validation. To that end, we identified 197 unmapped SSR markers that occurred within the 8 QTL intervals. These markers represent a potential source of SSR markers for use in saturating QTL intervals. Since QTLs identified in this study were independently verified in other studies, they potentially harbor key genes mediating biomass productivity as well as the expression of heterosis in *Populus* hybrids. The cumulative results presented in this study provide a basis for further genomic characterization of these high-value QTLs for subsequent use in improving biomass productivity in *Populus*.

## Materials and Methods

Experiments were conducted in field sites located in Boardman and Clatskanie, Oregon. These two sites contrast in water availability and provide opportunity for characterizing the influence of differential water availability in trait expression [10]. Details related to the mapping population, experimental design, and phenotypic measurements were described previously [12], [14], [15]. Briefly, an interspecific T×D hybrid population (F_1_ Family 53) was generated in 1981 from a cross between a female *P. trichocarpa* (T, black cottonwood clone 93–968) and a male *P. deltoides* (D, eastern cottonwood clone ILL-129). Two resulting hybrids (F_1_ clones 53–246 and 53–242, female and male, respectively) were sib-mated in 1988 and again in 1990 to generate an F_2_ mapping population, Family 331; of approximately 375 individuals [13], [15]. Phenotypic data for stem height and diameter for 310 of these individuals were reanalyzed in this study. Specifically, height and diameter data collected after 4 years of growth were reanalyzed for each site and height data collected after 8 years of growth was reanalyzed for the Clatskanie site.

### Genomic Resources

We used the family 331 and *Populus* consensus genetic maps described by Yin et al. [21], [25] for QTL identification and genome anchoring. Briefly, the family 331 map was based on 841 AFLP, RAPD, RFLP and SSR markers. Of these, 155 SSR markers were shared with the consensus map and were used to align the family 331 map to the 19 LGs corresponding to the *Populus* haploid chromosome number. Detailed map characteristics, marker nomenclature, and the resulting syntenic relationship between the genetic maps were summarized in Yin et al. [21].

Additionally, we used 2,524 SSR markers with known positions on the *Populus* V2.2 genome assembly (http://www.phytozome.net/cgi-bin/gbrowse/poplar/) to establish the genome assembly framework used for anchoring QTL. Procedure for assigning SSR markers to physical positions on the genome assembly was previously described by Ranjan et al. [20]. Briefly, the physical position of SSR markers in *Populus* genome sequence was obtained by BLAST search of the corresponding forward and reverse primers. Additional checks were done to ensure that the predicted SSR length based on BLAST result was same as the length of the actual sequenced SSR marker. MapChart 2.2 [26] was used to graphically represent synteny and collinearity between the genetic maps and the genome assembly.

### QTL Analysis

The Multiple-QTL Model (MQM) package of MapQTL 6.0 [27] with automatic cofactor selection was used to map putative and suggestive QTL intervals on the family 331 genetic map. The non-parametric Kruskal-Wallis (KW) analysis with a significance threshold of 0.005 was used as secondary confirmation of detected QTL. Mean phenotypic values across ramets and replicates were analyzed separately for each trait and experiment. The criteria for declaring QTL was based on the MQM analysis in which results were subjected to permutation tests [28]. 1000 permutations were conducted separately for each trait and experiment to determine linkage groupwise and genomewise LOD significance thresholds at the 0.05 significance level. A putative QTL was declared when it was detected in at least two experiments or in the same experiment for both traits, with at least one of those instances exceeding the linkage groupwise LOD significance threshold. A suggestive QTL was declared when detected in at least two experiments or in one experiment for both traits, with LOD scores above 2.0 but failing to exceed the LOD significance threshold in any one instance [29]. Results of the KW analysis were used for QTL verification only and were not reported in this manuscript. RAPDS and RFLP markers were excluded in the QTL analysis due to the small population size with available genotypic information for these markers as described by Yin et al. [21]. Nomenclature for naming putative biomass QTL was based on the abbreviation *BM* (biomass) and were numbered according to increasing LG number.

### QTL x Environment Interaction

Effects of environment on QTL detection were estimated using the Additive Main Effects and Multiplicative Interaction Model (AMMI) approach [30]. In order to verify QTL-specific changes in response to environment, LOD scores for individual markers within a QTL interval were used in the analysis with environment being the Boardman and Clatskanie sites and each experiment being considered as a replicate for each respective site. Only markers falling within 1-LOD difference on either side of the QTL peak were selected for analysis. The AMMI model equation was:
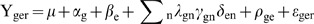
Where Y*_ger_* = the observed LOD score of g^th^ genotype (marker) in e^th^ environment for r^th^ replicate;

µ = grand mean; α*_g_* = deviation of mean of g^th^ marker from grand mean; β_e_ = deviation of mean of the e^th^ environment from the grand mean; λ_gn_ = the singular value for the n^th^ interaction principal component axis (PCA); γ_gn_ = the marker eigenvector for the n^th^ PCA; δ_en_ = the environment eigenvector values for the n^th^ PCA; ρ*_ge_* = residual effects; and ε*_ger_* = the error term.

### Additive and Dominant Effects

Locus specific additive and dominant effects were calculated from mean phenotypic values of the alternate homozygous genotypes and heterozygotes at each QTL. Specifically, locus-specific phenotypic means for heterozygous loci carrying alleles derived from the same species (mu(ac), mu(bd)) and for heterozygous loci carrying alleles derived from both species (mu (ad), mu (bc)) were computed from the MQM mapping procedure using MapQTL 6.0 [27]. Additive (a), dominance (d) effects were calculated as:
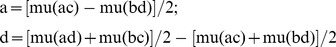
(27)


QTL mode of action was calculated as the ratio of dominance over additivity, d/|a| [14], [31]. d/|a|ratios less than 1 were regarded as reflecting under-dominance, ratios between 0 and 1 reflected partial dominance, and ratios greater than 1 reflected over-dominance as suggested by Hua et al. [31].

### Genome Anchoring of QTL Intervals

We used the genome anchoring strategy described by Ranjan et al. [20] with minor variation to establish QTL intervals on the genome assembly. In addition to establishing QTL intervals, we also estimated the physical position of the marker closest to the QTL peak within the QTL interval. In this regard, we used the cM to physical distance ratio determined from SSR markers flanking the QTL to calculate the approximate position of the QTL peak relative to their cM distance from each flanking SSR marker. Where more than one marker was associated with the QTL peak in different experiments, the cM position of the marker with most frequent peak association or the marker with the highest LOD score was used to estimate the QTL peak position on the genome assembly.

### Candidate Gene Identification and Characterization

Gene models lying within genome-anchored QTL intervals were identified from the *Populus* genome assembly V2.2 in the Phytozome database (http://www.phytozome.net/cgi-bin/gbrowse/poplar/). Annotations based on PFAM domains were used to exclude gene models with unknown function from the analysis and to establish duplication relationships between genes occurring in different QTL intervals. Paralogous relationships were verified based on information available for each gene model in the GRAMENE database (http://www.gramene.org).

## Supporting Information

Table S1 **SSR markers mapping in QTL intervals based on physical positions on the** ***Populus*** **genome assembly.**
(XLSX)

Table S2 ***Populus*** **gene models within QTL intervals identified for stem height and diameter in an F_2_ pedigree.**
(XLSX)

Table S3 **Gene duplication within and between QTL intervals identified from stem height and diameter in an F_2_** ***Populus*** **pedigree.**
(XLSX)

## References

[1] Ranney JW, Wright LL, Layton PA (1987) Hardwood energy crops: The technology of intensive culture. Journal of Forestry 85: 17–28.

[2] Abelson PH (1991) Improved yields of biomass. Science 252: 1469. 17834850 10.1126/science.252.5012.1469

[3] Zsuffa L, Giordano E, Pryor LD, Stettler RF (1996) Trends in poplar culture: some global and regional perspectives. In: Stettler RF, Bradshaw HD Jr, Heilman PE, Hinckley TM eds. Biology of Populus and its implications for management and conservation. Ottawa, ON, Canada: NRC Research Press, National Research Council of Canada. Pp. 515–539.

[4] Tuskan GA (1998) Short-rotation woody crop supply systems in the United States: what do we know and what do we need to know? Biomass Energy 14: 307–315.

[5] Studer MH, DeMartini JD, Davis MF, Sykes RW, Davison B, et al (2011) Lignin content in natural *Populus* variants affects sugar release. Proceedings of the National Academy of Sciences 108: 6300–6305. 10.1073/pnas.1009252108 PMC3076829 21444820

[6] Zhuang Y, Adams KL (2007) Extensive allelic variation in gene expression in *Populus* F_1_ hybrids. Genetics 177: 1987–1996. 18073418 10.1534/genetics.107.080325 PMC2219518

[7] Eckenwalder JE (2001) Descriptions of clonal characteristics In: Dickmann DI, Isebrands JG, Eckenwalder JE, Richardson J eds. (2001) Poplar culture in North America 2001. Ottawa, ON, Canada: NRC Research Press, National Research Council of Canada. 331–382.

[8] Scarascia-Mugnozza GE, Ceulemans R, Heilman PE, Isebrands JG, Stettler RF, et al (1997) Production physiology and morphology of *Populus* species and their hybrids grown under short rotation II Biomass components and harvest index of hybrid and parental species clones. Canadian Journal of Forestry Research 27: 285–294.

[9] Stettler RF, Wu R, Zsuffa L (1996) The role of hybridization in the genetic manipulation of *Populus* In: Stettler RF, Bradshaw HD Jr, Heilman PE, Hinckley TM eds. Biology of Populus and its implications for management and conservation. Ottawa, ON, Canada: NRC Research Press, National Research Council of Canada. 87–112.

[10] Tschaplinski TJ, Tuskan GA, Sewell MM, Gebre GM, Todd DE, et al (2006) Phenotypic variation and QTL identification for osmotic potential in an interspecific hybrid inbred F_2_ poplar pedigree growing under contrasting environments. Tree Physiology 26: 595–604. 16452073 10.1093/treephys/26.5.595

[11] Rae AM, Pinel MPC, Bastien C, Sabatti M, Street NR, et al (2008) QTL for yield in bioenergy Populus: identifying GxE interactions from growth at three contrasting sites. Tree Genetics and Genomes 4: 97–112.

[12] Wu R (1997) Genetic control of macro- and micro-environmental sensitivities in *Populus* . Theoretical Applied Genetics 94: 104–114. 19352752 10.1007/s001220050388

[13] Bradshaw HD Jr, Villar M, Watson BD, Otto KG, Stewart S, et al (1994) Molecular genetics of growth and development in *Populus* III A genetic linkage map of a hybrid poplar composed of RFLP, STS, and RAPD markers. Theoretical Applied Genetics 89: 167–178. 24177824 10.1007/BF00225137

[14] Bradshaw HD Jr, Stettler RF (1995) Molecular genetics of growth and development in *Populus* IV Mapping QTLs with large effects on growth, form, and phenology traits in a forest tree. Genetics 139: 963–973. 7713445 10.1093/genetics/139.2.963 PMC1206394

[15] Wu R, Stettler RF (1997) Quantitative genetics of growth and development in *Populus* II The partitioning of genotype × environment interaction in stem growth. Heredity 78: 124–134.

[16] Wullschleger SD, Yin TM, DiFazio SP, Tschaplinski TJ, Gunter LE, et al (2005) Phenotypic variation in growth and biomass distribution for two advanced-generation pedigrees of hybrid poplar. Canadian Journal of Forest Research 35: 1779–1789.

[17] Novaes E, Osorio L, Drost DR, Miles BL, Boaventura-Novaes CRD, et al (2009) Quantitative genetic analysis of biomass and wood chemistry of *Populus* under different nitrogen levels. New Phytologist 182: 878–890. 19291008 10.1111/j.1469-8137.2009.02785.x

[18] Tuskan GA, DiFazio S, Jansson S, Bohlmann J, Grigoriev I, et al (2006) The genome of black cottonwood, *Populus trichocarpa* (Torr & Gray). Science 313: 1596–1604. 16973872 10.1126/science.1128691

[19] Slavov GT, DiFazio SP, Martin J, Schackwitz W, Muchero W, et al. (2012) Genome resequencing reveals multiscale geographic structure and extensive linkage disequilibrium in the forest tree *Populus trichocarpa*. New Phytologist. doi: 10.1111/j.1469-8137.2012.04258.x. 10.1111/j.1469-8137.2012.04258.x 22861491

[20] Ranjan P, Yin T, Zhang X, Kalluri UC, Yang X, et al (2010) Bioinformatics-Based Identification of Candidate Genes from QTLs Associated with Cell Wall Traits in *Populus.* . Bioenergy Research 3: 172–182.

[21] Yin T, Zhang X, Gunter L, Ranjan P, Sykes R, et al (2010) Differential detection of genetic loci underlying stem and root lignin content in *Populus.* . Plos One 5: e14021. 21151641 10.1371/journal.pone.0014021 PMC2999904

[22] Brown GR, Bassoni DL, Gill GP, Fontana JR, Wheeler NC, et al (2003) Identification of quantitative trait loci influencing wood property traits in loblolly pine (*Pinus taeda* L) III QTL verification and candidate gene mapping. Genetics 164: 1537–1546. 12930758 10.1093/genetics/164.4.1537 PMC1462646

[23] Muchero W, Ehlers JD, Roberts PA (2010) Restriction site polymorphism-based candidate gene mapping for seedling drought tolerance in cowpea [*Vigna unguiculata* (L.) Walp.]. Theoretical Applied Genetics 120: 509–518. 19834655 10.1007/s00122-009-1171-6 PMC2807941

[24] Muchero W, Ehlers JD, Close TJ, Roberts PA (2011) Genic SNP markers and legume synteny reveal candidate genes underlying QTL for *Macrophomina phaseolina* resistance and maturity in cowpea [*Vigna unguiculata* (L) Walp]. BMC Genomics 12: 8. 21208448 10.1186/1471-2164-12-8 PMC3025960

[25] Yin T, DiFazio SP, Gunter LE, Zhang X, Sewell MM, et al (2008) Genome structure and emerging evidence of an incipient sex chromosome in *Populus.* . Genome Research 18: 422–430. 18256239 10.1101/gr.7076308 PMC2259106

[26] Voorrips RE (2002) MapChart: Software for the graphical presentation of linkage maps and QTLs. Heredity 93: 77–78. 10.1093/jhered/93.1.77 12011185

[27] Van Ooijen JW (2009) MapQTL ® 6, Software for the mapping of quantitative trait loci in experimental populations of diploid species. Kyazma BV, Wageningen, Netherlands.

[28] Churchill GA, Doerge RW (1994) Empirical threshold values for quantitative trait mapping. Genetics 138: 963–971. 7851788 10.1093/genetics/138.3.963 PMC1206241

[29] Wu R, Bradshaw HD, Stettler R (1997) Molecular Genetics of Growth and Development in *Populus* (Salicaceae). V. Mapping Quantitative Trait Loci Affecting Leaf Variation. American Journal of Botany 84: 143–153. 21712194

[30] Gauch HG (1988) Model selection and validation for yield trials with interactions. Biometrics 44: 705–715.

[31] Hua J, Xing Y, Wu W, Xu C, Sun X, et al (2003) Single-locus heterotic effects and dominance by dominance interactions can adequately explain the genetic basis of heterosis in an elite rice hybrid. Proceedings of the National Academy of Sciences 100: 2574–2579. 10.1073/pnas.0437907100 PMC151382 12604771

